# Development of soft tissue asymmetry indicators to characterize aging and functional mobility

**DOI:** 10.3389/fbioe.2023.1282024

**Published:** 2023-12-12

**Authors:** Carlo Ricciardi, Alfonso Maria Ponsiglione, Marco Recenti, Francesco Amato, Magnus Kjartan Gislason, Milan Chang, Paolo Gargiulo

**Affiliations:** ^1^ Department of Electrical Engineering and Information Technology, University of Naples “Federico II”, Naples, Italy; ^2^ Institute of Biomedical and Neural Engineering, Reykjavik University, Reykjavik, Iceland; ^3^ The Icelandic Gerontological Research Institute, Landspitali University Hospital, Reykjavik, Iceland; ^4^ Department of Science, Landspitali University Hospital, Reykjavik, Iceland

**Keywords:** aging, asymmetry, CT scan, body mass index, gait speed, soft tissue, muscle, sarcopenia

## Abstract

**Introduction:** The aging population poses significant challenges to healthcare systems globally, necessitating a comprehensive understanding of age-related changes affecting physical function. Age-related functional decline highlights the urgency of understanding how tissue composition changes impact mobility, independence, and quality of life in older adults. Previous research has emphasized the influence of muscle quality, but the role of tissue composition asymmetry across various tissue types remains understudied. This work develops asymmetry indicators based on muscle, connective and fat tissue extracted from cross-sectional CT scans, and shows their interplay with BMI and lower extremity function among community-dwelling older adults.

**Methods:** We used data from 3157 older adults from 71 to 98 years of age (mean: 80.06). Tissue composition asymmetry was defined by the differences between the right and left sides using CT scans and the non-Linear Trimodal Regression Analysis (NTRA) parameters. Functional mobility was measured through a 6-meter gait (Normal-GAIT and Fast-GAIT) and the Timed Up and Go (TUG) performance test. Statistical analysis included paired t-tests, polynomial fitting curves, and regression analysis to uncover relationships between tissue asymmetry, age, and functional mobility.

**Results:** Findings revealed an increase in tissue composition asymmetry with age. Notably, muscle and connective tissue width asymmetry showed significant variation across age groups. BMI classifications and gait tasks also influenced tissue asymmetry. The Fast-GAIT task demonstrated a substantial separation in tissue asymmetry between normal and slow groups, whereas the Normal-GAIT and the TUG task did not exhibit such distinction. Muscle quality, as reflected by asymmetry indicators, appears crucial in understanding age-related changes in muscle function, while fat and connective tissue play roles in body composition and mobility.

**Discussion:** Our study emphasizes the importance of tissue asymmetry indicators in understanding how muscle function changes with age in older individuals, demonstrating their role as risk factor and their potential employment in clinical assessment. We also identified the influence of fat and connective tissue on body composition and functional mobility. Incorporating the NTRA technology into clinical evaluations could enable personalized interventions for older adults, promoting healthier aging and maintaining physical function.

## Introduction

The rapidly growing older population has become a significant healthcare concern in most developed societies (Kalseth and Halvorsen, 2020). With advances in healthcare and technology, people are living longer and healthier lives, but there is an urgent need to develop strategies to address the unique challenges of aging, including health disparities, social isolation, and financial insecurity (WHO, 2020). One of the most prominent manifestations of aging is functional decline, resulting in decreased muscle strength, an increased risk for falls, gait and balance problems, and chronic pain which highly affects the independency of older adults (Chatterjee et al., 2021).

Mobility, which is defined as the ability to move without assistance (Ostir et al., 2015; Treacy et al., 2022) is crucial for older adults to manage independent daily life (Visser et al., 2005; Schaap et al., 2013). Lower extremity function (LEF) is a critical measure of mobility and is frequently used as a clinical screening tool (Guralnik et al., 2000; Chang et al., 2013). The age-related decline in muscle mass and strength (Visser et al., 2005; Schaap et al., 2013) impacts the ability to walk quickly and efficiently in older adults, which ultimately leading to a slower gait speed over time (Goodpaster et al., 2006; Schaap et al., 2013; Reinders et al., 2015).

Sarcopenia, which refers to the simultaneous decline in skeletal muscle size and quality, has consistently been associated with various pathophysiological mechanisms that ultimately result in reduced lean tissue mass and the gradual fat infiltration of non-contractile tissue into lean muscle. This condition increases the risk of disability and mortality (Young et al., 1985; Kalyani et al., 2014). In older adults, there is often an asymmetrical distribution of muscle volume accompanied by a decrease in muscle mass and strength (Stagi et al., 2021). The asymmetry in muscle volume, particularly in the lower extremities, has been identified as a contributing factor to slower walking speed among older adults (Laroche et al., 2012; Lee et al., 2019; Mertz et al., 2019). Previous research has shown that older adults with asymmetrical muscle and fat mass exhibit poor physical performance in areas such as gait speed, balance, strength, and flexibility (Laroche et al., 2012; Miller et al., 2015; Lee et al., 2019; Mertz et al., 2019). These studies assessed muscle and fat mass asymmetry using techniques such as dual-energy X-ray absorptiometry (DEXA) (Lee et al., 2019; Mertz et al., 2019), computed tomography (CT) (Miller et al., 2015), and muscle strength measurements (Laroche et al., 2012). While existing studies have primarily focused on assessing muscle asymmetry using techniques such as dual-energy X-ray absorptiometry (DEXA), computed tomography (CT), and muscle strength measurements, there is a gap in the literature regarding asymmetry in other soft tissues of the legs, such as fat or connective tissue.

In recent years, our research group has focused on studying sarcopenia and has developed an approach based on analyzing features extracted from the radiodensitometric distribution of x-ray CT scans of mid-thigh cross-sectional images. This approach is known as the non-linear trimodal regression analysis (NTRA) (Edmunds et al., 2016; Edmunds et al., 2018). The NTRA features, consisting of 11 patient-specific parameters that characterize the quantity and quality of muscle, fat, and connective tissues using Hounsfield Unit (HU) radiodensitometric values, have shown predictive potential in classifying comorbidities such as diabetes and hypertension using Machine Learning (ML) in the older population (Recenti et al., 2020a). Additionally, ML algorithms have been employed in conjunction with the NTRA features to classify cardiac pathophysiologies, Body Mass Index (BMI), and isometric leg strength, achieving classification metrics with accuracy rates exceeding 95% (Ricciardi et al., 2020a; Recenti et al., 2020b; Recenti et al., 2021a). These features have also mediated the relationship between physical activity and lower extremity function (LEF) in aging individuals (Edmunds et al., 2021). Furthermore, previous studies observed that muscle features exhibit more significant variations compared to fat and connective tissues with regard to age and physical activity levels (Recenti et al., 2021b).

Despite the growing recognition of the importance of muscle quality and mobility (Simonsick et al., 2008; Reinders et al., 2015; Gonzalez et al., 2020; Borghi et al., 2022; Khaleghi et al., 2023), there still exists a lack of studies examining asymmetry and its implications regarding mobility in older adults. In contrast to previous studies focusing solely on NTRA features for one leg, a new approach assessing asymmetries in NTRA features may provide insight into how asymmetry in these three tissue types in the thigh relates to age and lower extremity function (LEF) among older adults. Furthermore, it may offer potential implications for the clinical assessment of age-related or changes in muscle quality and mobility among older adults (Harris et al., 2007). The aim of the current study is to investigate the differences between right and left legs using the NTRA features, establishing a set of asymmetry indicators, and assessing their relationships with age, BMI, gait speed and time up and go (TUGO). Data was from the large cohort of the AGES-Reykjavik study based on community-dwelling older adults in Iceland (Harris et al., 2007).

## Materials and methods

### Ages-Reykjavik dataset

The AGES-Reykjavik dataset includes, for the present study, a total of 3157 healthy elderly subjects from 71 to 98 years of age (mean: 80.06). All the participants were measured in a series of multimetric assessments including CT-scans, BMI and LEF performance tests (Harris et al., 2007). Informed signed consent was given by all participants. The ethical approval was certified by the Icelandic National Bioethics Committee (RU Code of Ethics, Paragraph 3—Article 2—Higher Education Institution Act 63/2006). A limited number of subjects was not able to complete one or more gait performance test due to different reasons so the total number of subjects considered for each of the next measurements can be lower than 3157 because of the presence of missing values in the dataset.

### Anthropometric data

Heigh and weight of each subject were objectively measured (Chang et al., 2013; Recenti et al., 2021a) and BMI was determined by dividing the weight in kilograms by the square of the height in meters.

For the asymmetry analysis four BMI classes have been defined as follows (Table 1) (WHO, 2021):

• Class 1: BMI <18.5: below-normal (N = 53)
• Class 2: 18.5 < BMI <25: normal (N = 1092)
• Class 3: 25 < BMI <30: above-normal (N = 1341)
• Class 4: BMI >30: overweight (N = 667)

**TABLE 1:** AGES Dataset: Sex differences and mean age in respect to BMI classes.

	BMI			
	<18.5	18.5–25	25–30	>30
Subject, N	53	1092	1341	667
Age, yrs, Mean (SD)	76.81 (5.98)	75.44 (5.97)	74.73 (5.96)	73.77 (5.96)
Male, N	21	466	561	269
Female, N	32	626	780	398

### LEF performance tests

To assess the LEF, two tests were conducted: the 6-meter walk test and the Timed Up and Go Test (TUG) (Chang et al., 2013). The 6-meter gait walk test included two measurements, normal-GAIT and fast-GAIT, in meters per second (m/s). The test is reliable when performed standardly and well tolerated by elderly individuals (Cesari et al., 2005). Two gait speed data were consequently extracted: Normal Gait Speed and Fast Gait Speed. The normal gait has been proved to be strongly linked to disability, risk of fall and mortality (Guralnik et al., 2000; Studenski et al., 2011; Nakakubo et al., 2018) while the fast gait speed represents the maximum walking speed of older individual which is used as a predictor of frailty, falls, and mobility limitation (White et al., 2013).

The normal-GAIT Task, two classes have been defined using a threshold based on the distribution of the speed (equal to 1 m/s) (defined as *v_norm*) to carry out the task and adapted from the literature (Day and Lord, 2018) and reported as follows:

• Class 1: *v_norm* < 1 m/s: slow (N = 1966)
• Class 2: *v_norm* > 1 m/s: normal (N = 1084)

Similarly, for the fast-GAIT Task two classes have been defined using a threshold based on the distribution of the speed 1.3 m/s (defined as *v_fast*) to carry out the task and adapted from the literature (Day and Lord, 2018) and reported as follows:

• Class 1: *v_fast* < 1.3 m/s: slow (N = 1957)
• Class 2: *v_fast* > 1.3 m/s: normal (N = 800)

The TUG measured the time in seconds, it took for participants to stand up from a seated position (height of the chair: 45.5 cm), walk a distance of 3 m, turn around, walk back to the chair, and sit down again (Podsiadlo and Richardson, 1991). TUG is a valuable screening tool for detecting balance issues in older adults and is also used as a predictor of decline in daily activities (Newton, 1997). During the test, participants were allowed to wear their own footwear, and if needed, a cane or walker could be used. The time recorded for the first complete trial was used for the analysis (Chang et al., 2013).

The TUG has been defined in four classes using thresholds based on the distribution of the time to carry out the task and adapted from the literature (Podsiadlo and Richardson, 1991) and reported as follows:

• Class 1: TUG <10 s: fast (N = 660)
• Class 2: 10 s < TUG <13 s: average (N = 1333)
• Class 3: 13 s < TUG <29 s: slow (N = 1044)
• Class 4: TUG >29 s: very slow (N = 16)

Table 2 shows the dataset population age and sex characteristics with a focus on the LEF tests and their classes.

**TABLE 2:** AGES Dataset: Sex differences and mean age in respect to LEF tests.

	Normal-GAIT		Fast-GAIT		TUG			
	<1 m/s	>1 m/s	<1.3 m/s	>1.3 m/s	<10 s	10–13 s	13–29 s	>29 s
Subject, N	1966	1084	1957	800	660	1333	1044	16
Age, yrs Mean (SD)	79.97 (4.90)	80.04 (4.98)	80.05 (4.86)	79.92 (4.94)	79.91 (4.95)	80.05 (4.85)	80.14 (4.90)	80.06 (5.68)
Male, N	821	456	812	352	298	553	423	8
Female, N	1145	628	1145	448	362	780	621	8

### CT acquisition

All individuals included in the AGES-Reykjavík database underwent scanning using a 4-row CT detector system with a voltage setting of 120-kV (Sensation; Siemens Medical Systems, Erlangen, Germany). The scanned area spanned from the iliac crest to the knee. Prior to transaxial imaging, accurate positions were established by measuring the maximum length of the femur on an anterior-posterior localizer image, followed by locating the midpoint of the femoral long axis. Subsequent to CT image acquisition, a single 10 mm section was extracted from the middle of the thigh for each participant, positioned equidistantly between the acetabulum of the hip joint and the knee joint (Harris et al., 2007). The pixel elements within each slice were then subjected to processing using the NTRA method, enabling the derivation of personalized radiodensitometric value distributions ranging from −200 to 200 HU.

### Non-linear trimodal regression analysis (NTRA) and asymmetry indicators

As mentioned previously, this study utilized the NTRA method to computationally characterize each distribution of HU. The NTRA method, originally described in the study by Edmunds et al., 2016, treats each HU distribution as a quasi-probability density function that is defined by three Gaussian distributions. This definition arises from the hypothesis that HU distributions of cross-sectional soft tissues exhibit three distinct peaks or modes, corresponding to three separate tissue types with their own specific ranges of linear attenuation coefficients in the HU domain. These tissue types and their corresponding HU ranges are as follows: adipose or fat tissue [-200 to −10 HU], loose connective tissue [-9 to 40 HU], and lean muscle [41 to 200 HU]. The general form of this quasi-probability density function, representing the trimodal nature, can be expressed as Eq. 1, where N denotes the amplitude, μ represents the location, and σ indicates the width of the distribution. The parameter α captures the asymmetry (skewness) of the fat and muscle distributions, with a value of zero (non-skewed) assigned to the central connective tissue distribution.

$$\sum_{i=1}^{3}\varphi \left(x,N_{i},\mu_{i},\sigma_{i},\alpha_{i}\right)=\sum_{1}^{3}\frac{N_{i}}{\sigma_{i}\sqrt{2\pi }}e^{-\frac{{\left(x-\mu_{i}\right)}^{2}}{2{\sigma_{i}}^{2}}}erfc\left(\frac{\alpha_{i}\left(x-\mu_{i}\right)}{\sigma_{i}\sqrt{2}}\right)$$ (1)

**FIGURE 1:**
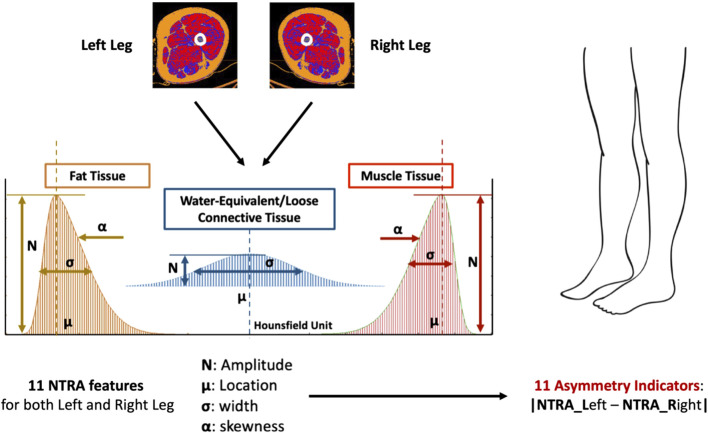
The 11 NTRA features are extracted from CT scan of both legs for a total of 22 NTRA features per subject. The Asymmetry Indicators are then computed as the absolute value of the NTRA difference between Left and Right legs. Each subject will have a total of 11 Asymmetry Indicators.

By employing a generalized reduced gradient algorithm and minimizing the standard error at each radio-absorption bin, theoretical HU distribution curves were generated, enabling the extraction of a total of 11 subject-specific NTRA parameters, 4 (N, μ, σ, α) from fat and muscle and 3 (N, μ, σ) from connective tissue (Figure 1).

**FIGURE 2:**
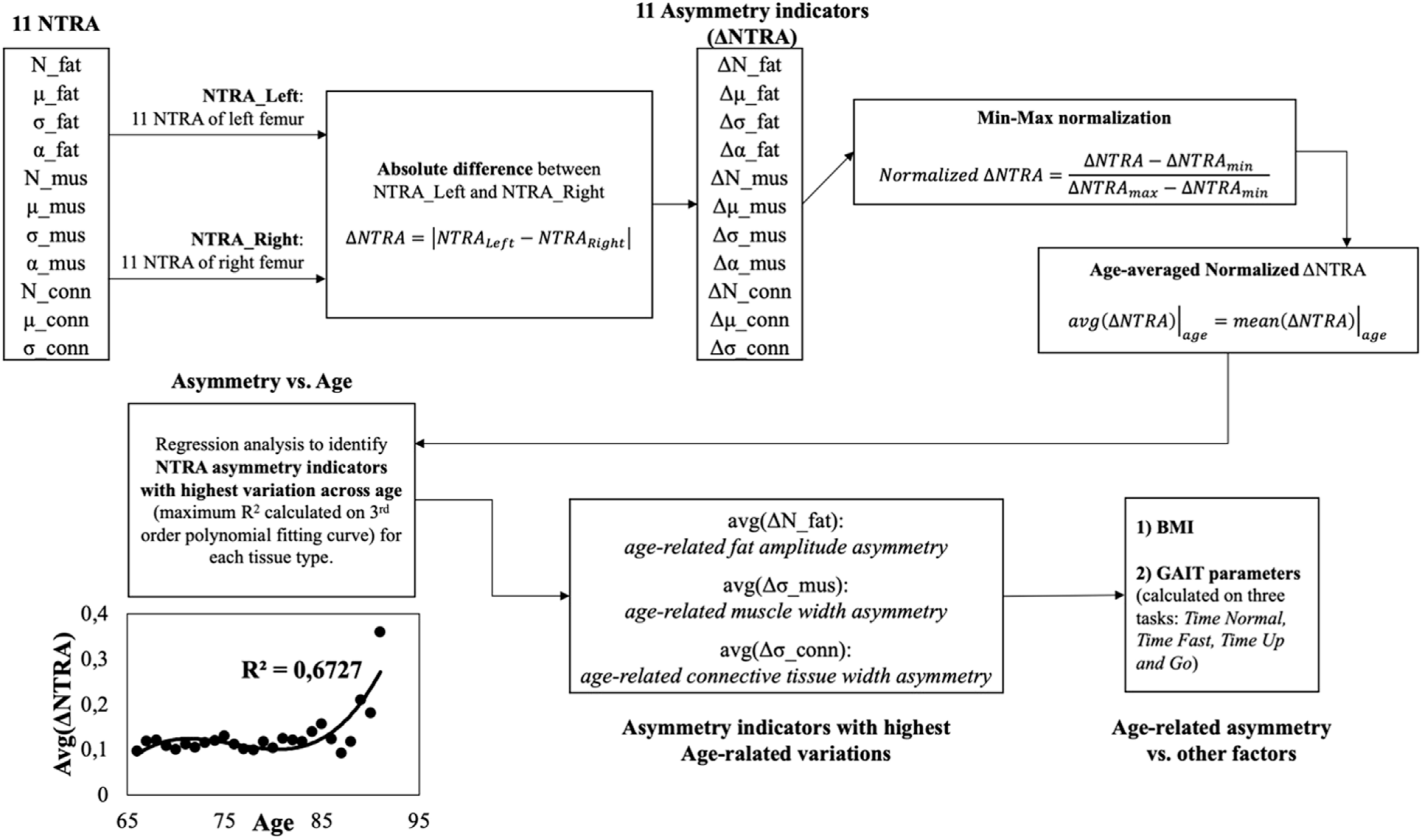
Workflow of the present study. Starting from the values of the 11 NTRA parameters for each leg (right ${{NTRA}_{Right}}_{i}$ and left ${{NTRA}_{Left}}_{i}$ ) (see block n.1 of Figure 2), the asymmetry indicators were calculated according to the following formula (see block n.2 of Figure 2)..

The Asymmetry Indicators are computed as the absolute value of the NTRA difference between the values extracted from the left and the right legs (Eq. 2). Consequently, a total of 11 NTRA Asymmetry values is evaluated for each subject (Figures 1, 2).

$$\left.∆\left.\text{NTRA}=\right|{\text{NTRA}}_{\text{Left}}-{\text{NTRA}}_{\text{Right}}\right|$$ (2)

### Statistical analysis

The first analysis to find an overall asymmetry was performed by running a paired *t*-test to compare right and left NTRA parameters of each patient. The uncertainty level was set at 0.05. This test is used to investigate the presence of a difference between the right and the left NTRA parameter of each patient.

Then, the analytical workflow shown in Figure 2 has been adopted and described in the following.

$$Aymmetry\;indicator\;based\;on\;NTRA\;parameter=\;{\Delta NTRA}_{i}\;=\;\left|{{NTRA}_{Left}}_{i}-{{NTRA}_{Right}}_{i}\right|$$

$$with\;i\;=1,2,\ldots .11\;indicating\;the\;i-th\;among\;the\;11\;NTRA\;indices\;\left(e.g.,{{NTRA}_{Left}}_{1}=N_{fat}\;of\;the\;left\;leg\right)$$

The results are 11 left-right leg asymmetry indicators, ${\Delta NTRA}_{i}$ , (see block n. 3 of Figure 2).

These resulted indicators have been normalized using the min-max method according to the following formula (see block n. 4 of Figure 2):

$$normalized\;{\Delta NTRA}_{i}=n{\Delta NTRA}_{i}=\frac{{\Delta NTRA}_{i}-\min\left({\Delta NTRA}_{i}\right)}{\max\left({\Delta NTRA}_{i}\right)-\min\left({\Delta NTRA}_{i}\right)}$$

Then the arithmetic mean of the normalized ${\Delta NTRA}_{i}$ for each age category has been calculated (see block n. 5 of Figure 2):

$$age-averaged\;asymmetry\;indicator={\left.avg\left({\Delta NTRA}_{i}\right)\right|}_{age=y}=\frac{1}{Ny}\sum_{k=1}^{Ny}{\left.n{\Delta NTRA}_{i}\right|}_{k}$$

$$\begin{array}{c} with\;y=65,66\ldots ,95=patient\;age\;\left(in\;years\right),Ny=number\;of\;patients\;with\;age=y,\\ {\left.n{\Delta NTRA}_{i}\right|}_{k}\;is\;the\;value\;of\;the\;i-th\;n\Delta NTRA\;beloging\;to\;the\;k-th\;patient\;with\;age=y. \end{array}$$

Afterwards, the age-averaged asymmetry indicators have been plotted against the age groups and a regression curve (by means of 3-rd order polynomial fitting curves) was adopted to model the asymmetry trends vs. age and to study the relationships between subjects’ age and the degree of asymmetry (see block n. 6 of Figure 2). Based on the coefficient of determination (*R* ^2^) of the fitting curves, the asymmetry indicator with highest determination coefficient was chosen for each tissue type (fat, muscle and connective tissue) and have been moved for further analysis (see block n. 7 of Figure 2).

Finally, the degree of asymmetry was analyzed by grouping the data according to other physiological parameters, i.e., BMI, normal gait speed, fast gait speed, TUG (see block n. 8 of Figure 2). All the analyses were conducted using IBM SPSS v.27.

## Results

### NTRA paired t-tests

Table 3 shows the results of the statistical analysis conducted on AGES dataset to compare the values of the right and the left NTRA parameter of each patient. A paired *t*-test was conducted to show the overall differences.

**TABLE 3:** Statistical analysis through a paired *t*-test to show the overall differences between right and left NTRA parameters per each tissue.

		Tissue types from the mid-thigh cross-sectional CT								
NTRA		Fat			Muscle			Connective tissue		
Parameter	Side	M	SD	*p*	M	SD	*p*	M	SD	*p*
**N**	L	135.16	69.92	0.004	184.84	42.06	0.008	90.05	25.10	0.009
	R	137.48	71.81		186.41	41.36		91.10	25.03	
**μ**	L	11.95	7.76	0.004	23.14	6.18	<0.001	44.61	10.15	0.024
	R	11.59	7.20		22.65	5.95		45.01	9.66	
**σ**	L	−106.22	7.47	0.079	61.71	3.37	0.055	−46.4	26.80	0.010
	R	−106.42	7.01		61.6	3.23		−45.3	26.06	
**α**	L	0.018	0.14	0.807	3.09	0.67	<0.001			
	R	0.019	0.17		3.15	0.63				

CT: computed tomography, N: amplitude, μ: location, σ: width, α: skewness, L: left, R: right M: mean; SD: standard deviation; *p*: *p*-value.

Table 3 shows that amplitude and location of each tissue were statistically significantly different (*p* < 0.005 for each parameter). Connective tissue had also a statistically significant difference in width (*p* = 0.01) and muscle had a statistically significant difference in skewness (*p* < 0.001) and a trend in width (*p* = 0.055). These results already provide with an idea of overall presence of asymmetry in the analyzed patients.

### Asymmetry vs. age

**FIGURE 3:**
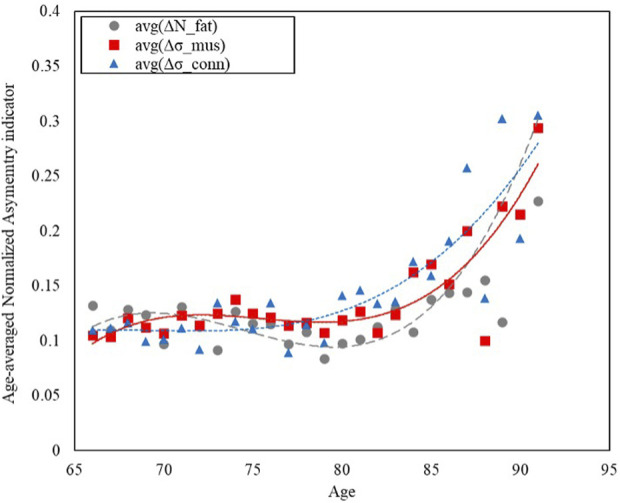
Trends of the age-averaged normalized values of the NTRA asymmetry indicators exhibiting most significant variation across population age for: Fat (avg(ΔN_fat_), grey circles), Muscle (avg(Δσ_mus_), red squares), and Connective Tissue (avg(Δσ_conn_), blue triangles). Third order polynomial fitting curves are also reported for each data series: Fat (avg(ΔN_fat_), grey dashed line), Muscle (avg(Δσ_mus_), red line), and Connective Tissue (avg(Δσ_conn_), blue dotted line).

By averaging the normalized asymmetry indicators (ΔNTRA) across the age groups and by fitting the age-related trends using a third order polynomial fitting curves, it was possible to determine the NTRA asymmetry indicators with the highest variation explained by the fitting model (i.e., highest determination coefficients, *R* ^2^) across age for each tissue type (fat, muscle, and connective tissue). The corresponding *R* ^2^ values are reported in Table 4 and the age-related trends for the three identified ΔNTRA with highest age-related variations are displayed in Figure 3.

**TABLE 4:** Determination coefficients of the third order polynomial fitting curves used (ΔNTRA) to fit the 11 age-averaged normalized values of the asymmetry indicators.

	Tissue types		
	Fat	Muscle	Connective tissue
NTRA parameter	*R* ^2^	*R* ^2^	*R* ^2^
avg (ΔN)	**0.50**	0.31	0.36
avg (Δμ)	0.23	0.34	0.21
avg (Δσ)	0.21	**0.76**	**0.75**
avg (Δα)	0.18	0.16	

N: amplitude, μ: location, σ: width, α: skewness, *R* ^2^: coefficient of determination.

Figure 3 shows how the asymmetry increases with age, with a growing trend that has been modelled with third order polynomial fitting curves for the three NTRA parameters exhibiting most significant variation across the population age for each tissue category, namely, N_fat_, σ_mus_, and σ_conn_.

### Asymmetry vs. BMI

**FIGURE 4:**
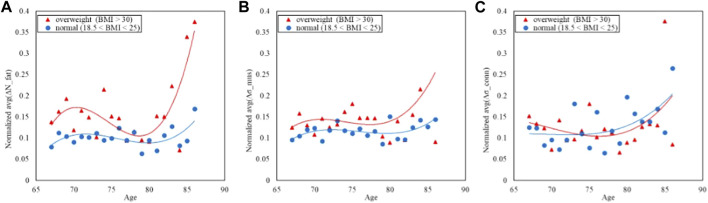
Trends of the age-averaged normalized values of ΔN_fat_ **(A)**, Δσ_mus_ **(B)**, and Δσ_conn_ **(C)** based on BMI groups, namely, overweight (red triangles) and normal (blue circles). Third order polynomial fitting curves are also reported for both overweight (red line) and normal (blue line) groups.

The values of the above selected NTRA asymmetry features have been investigated across the BMI groups as reported in Figure 4, following the four classes defined previously.

Figure 4 displays the comparison of the trends in the NTRA asymmetry indicators. The separability between normal and overweight BMI classes is far more evident in both the fat and muscle NTRA asymmetry parameters, where the average asymmetry is higher in the overweight group than the normal one. In the case of the connective tissue, the plot does not show appreciable separation among the two groups.

### Asymmetry vs. Gait

The study of potential confounding factors and covariates has been also carried out grouping the population according to the three LEF formerly defined (Gait Normal Task, Gait Fast Task and Gait TUG).

**FIGURE 5:**
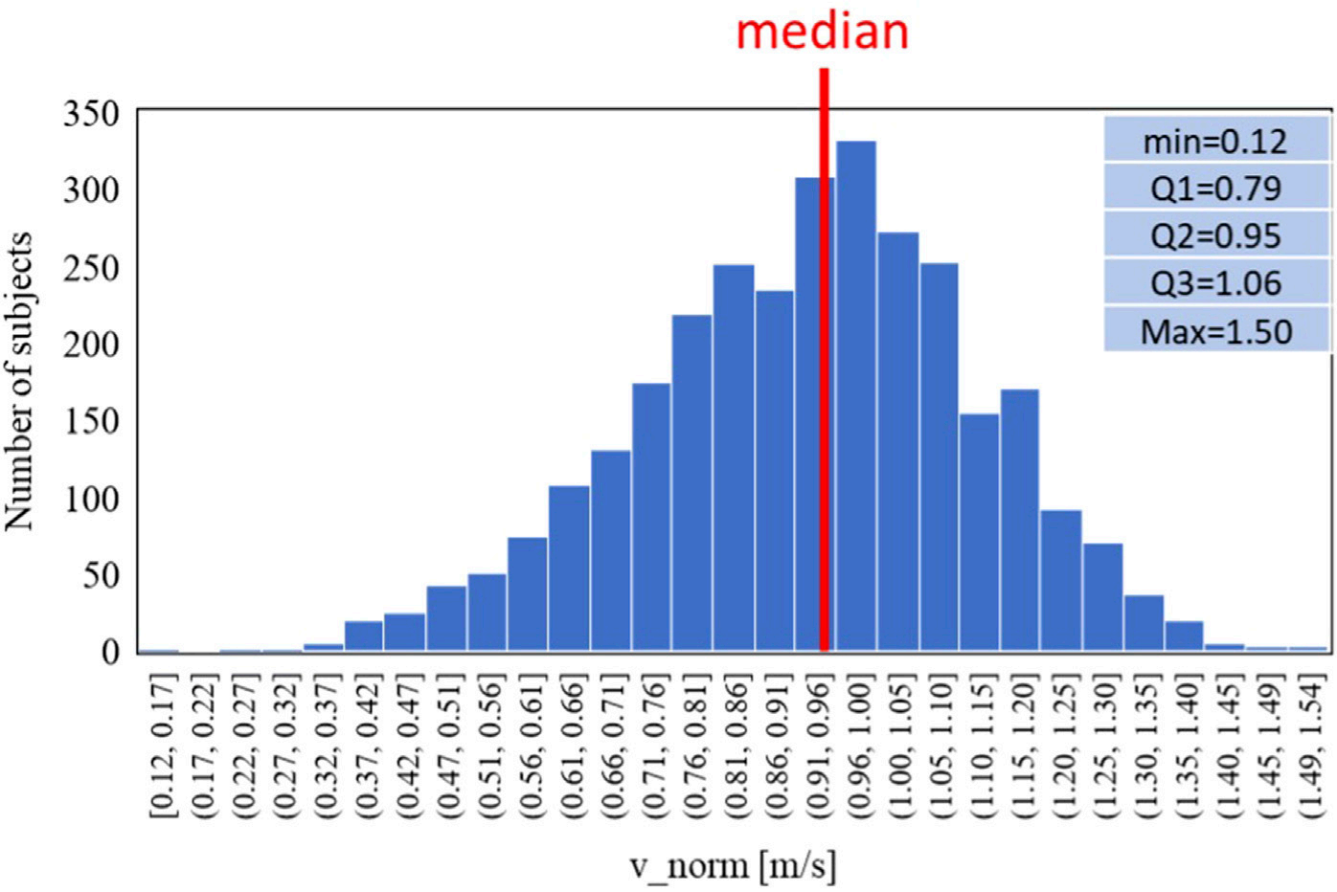
Data distribution of the normal-GAIT task reference parameter (*v_norm*).

**FIGURE 6:**
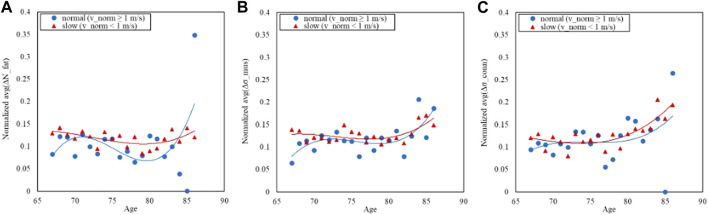
Trends of the age-averaged normalized values of ΔN_fat_ **(A)**, Δσ_mus_ **(B)**, and Δσ_conn_ **(C)** based on GAIT Normal Task groups, namely, slow (red triangles) and normal (blue circles). Third order polynomial fitting curves are also reported for both slow (red line) and normal (blue line) groups.

Figures 5, 6 refer to the normal-GAIT Task and report respectively the distribution of the speed to carry out the task (equal to 1 m/s) and the relative trends split according to the classes defined previously.

**FIGURE 7:**
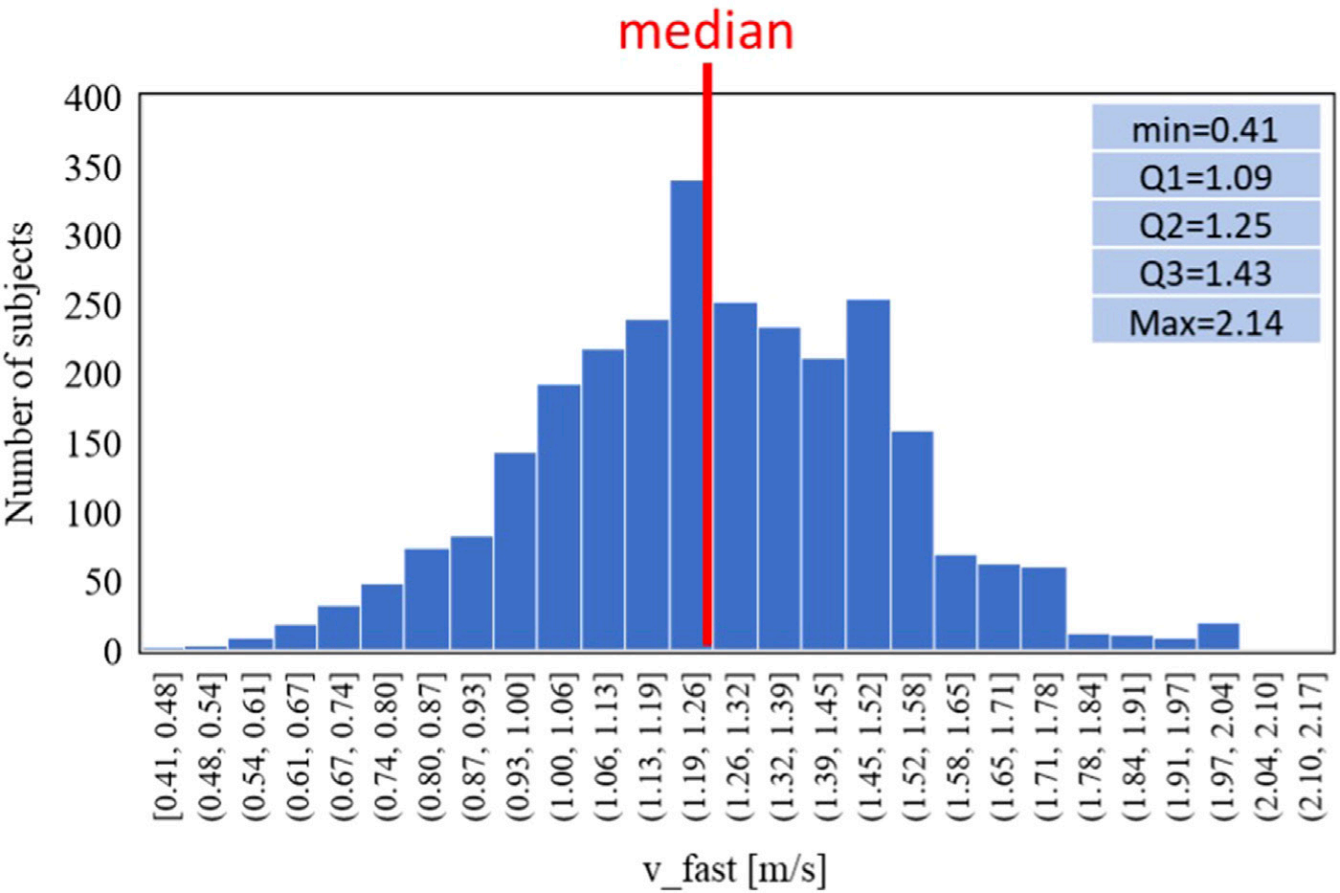
Data distribution of the fast-GAIT task reference parameter (*v_fast*).

**FIGURE 8:**
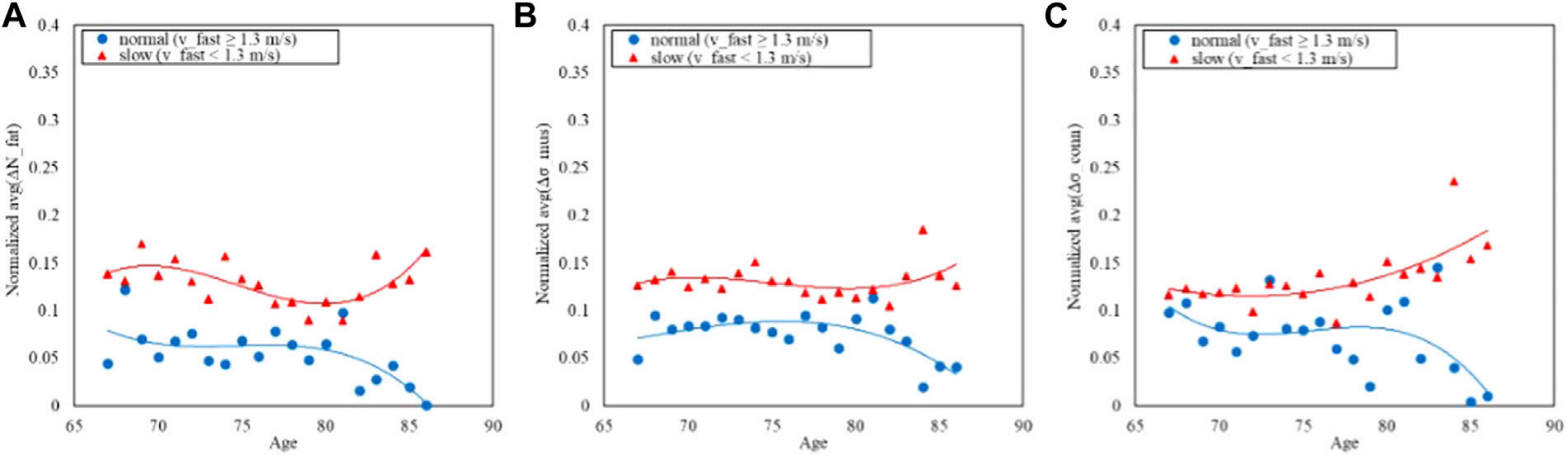
Trends of the age-averaged normalized values of ΔN_fat_ **(A)**, Δσ_mus_ **(B)**, and Δσ_conn_ **(C)** based on GAIT Fast Task groups, namely, slow (red triangles) and normal (blue circles). Third order polynomial fitting curves are also reported for both slow (red line) and normal (blue line) groups.

Similarly Figures 7, 8 report respectively the distribution of the speed in fast-GAIT (median 1.3 m/s) and the trends by a fast-GAIT Task.

**FIGURE 9:**
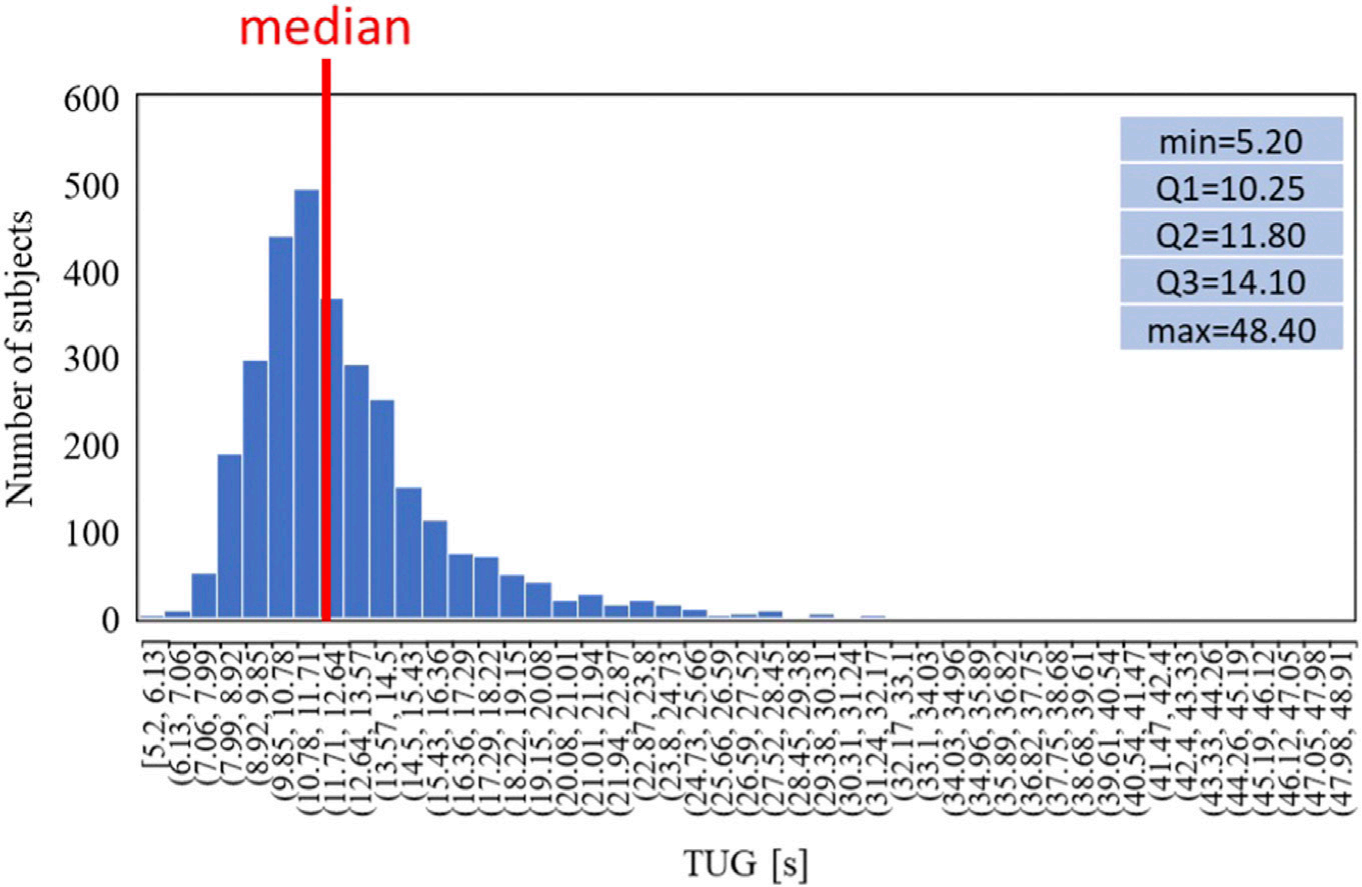
Data distribution of the TUG task.

**FIGURE 10:**
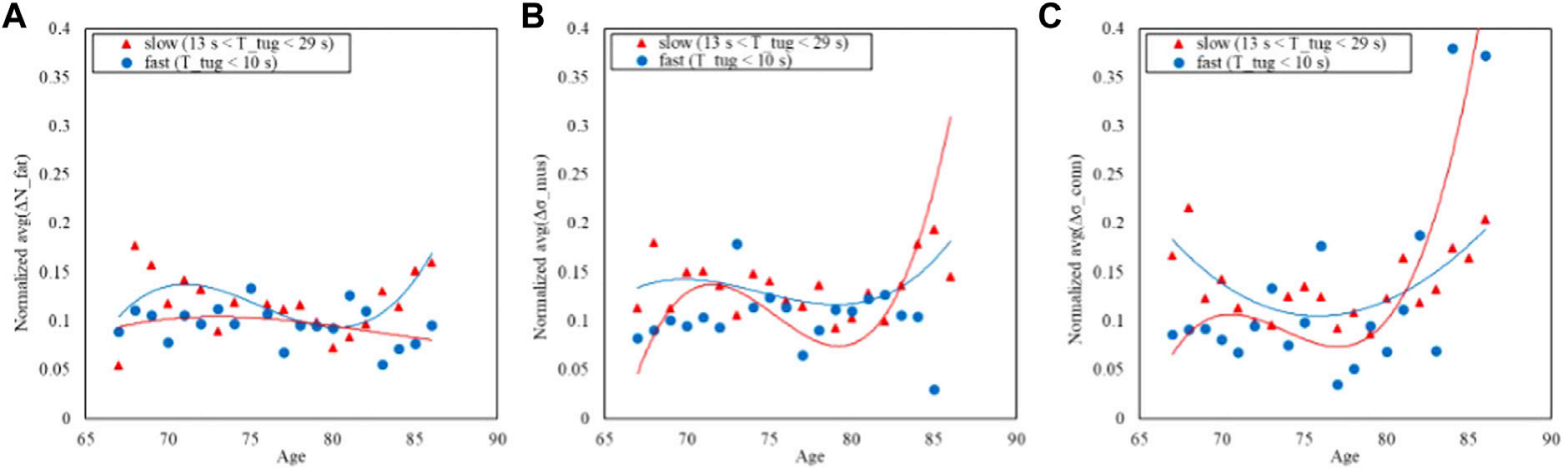
Trends of the age-averaged normalized values of ΔN_fat_ **(A)**, Δσ_mus_ **(B)**, and Δσ_conn_ **(C)** based on GAIT TUG groups, namely, slow (red triangles) and fast (blue circles). Third order polynomial fitting curves are also reported for both slow (red line) and normal (blue line) groups.

Finally, the same analysis is shown for the TUG: Figure 9 shows the distribution of the time while Figure 10 shows the trends split according to the TUG classes.

Figures 6, 8, 10 show how the Fast Task (Figure 8) can separate the normal and slow groups, thus confirming to be a confounding factor that could influence the asymmetry trend with age. On the contrary, Normal Task (Figure 6) and TUG (Figure 10) are not able to enhance the difference between the groups of subjects, thereby not showing a markable different trends of the asymmetry with age.

## Discussion

### Summary

This study aimed to explore whether there existed a connection between soft tissue composition asymmetry (heterogeneity), as defined by radiodensity analysis of thigh CT scans, and the functional mobility of the older Icelandic population. Our findings revealed that the asymmetry in tissue composition grows more pronounced with advancing age. Indeed, we observed that CT densitometric distribution can provide indicators of asymmetry in tissue composition (heterogeneity). The main results showed that tissue asymmetry increases with age with a nonlinear trend that could be modeled with third order polynomial curve to quantitatively describe up to 76% of the variation in the asymmetry across age. Moreover, our observations suggested that relationships between age and asymmetry could be also influenced by further possible confounding factors, such as BMI and mobility functions. The results also confirmed that the tissue composition asymmetry is a valuable indicator for evaluating age-related changes in muscle condition and provided insights for both musculoskeletal conditions and mobility function in older individuals. Particularly, three types of tissue composition including muscle, fat, and connective tissue provided comprehensive understanding of the intrinsic relationships between muscle quality, aging, and physical function. Recognizing the critical role of maintaining mobility function, especially among those at high risk of functional decline (Simonsick et al., 2008), our study underscores the importance of asymmetry information. Aligning with previous research (Wilkinson et al., 2018), our findings highlight that differences in muscle mass between the right and left thighs can result in imbalances affecting strength and function, particularly in movements like walking or climbing stairs. Variations in fat distribution impact body weight distribution, potentially influencing gait, and balance (Gonzalez et al., 2020; Khaleghi et al., 2023), while the connective tissue between muscle and fat in the thigh serves as a predictor of incident of mobility disability (Schaap et al., 2013; Reinders et al., 2015; Borghi et al., 2022). Despite the growing recognition of the importance of this asymmetry information, there is a notable absence of studies examining its implications in designing effective interventions to improve and sustain mobility function in older adults. Therefore, asymmetries in different tissue types can be a potential determinants of mobility limitation among older adults as well. In this context, the information regarding asymmetry in different tissue types in the thigh area can guide the targeted interventions and rehabilitation strategies to enhance the mobility.

### Asymmetry and physical function

Quantitative loss of muscle over age is a well-known condition in older adults (Curtis et al., 2015). Decline of muscle mass and function, defined as “sarcopenia,” is closely linked to impaired physical function and an increased risk of mortality in the aging population (Cawthon, 2015; Clark, 2019). Regarding the muscle aging in older adults, it is vital to recognize that the decline of muscle function is not only due to the loss of muscle mass but also other factors that affect the muscle quality including muscle composition and fatty infiltration (Goodpaster et al., 2006; Reinders et al., 2015). The asymmetry condition in the muscle strength among older adults was also reported in few studies (Skelton et al., 2002; Perry et al., 2007), and one recent study reported that asymmetry in lower extremity strength was associated with functional mobility among older community-living older adults (Lee et al., 2019).

### Muscle quality and physical function

The aging process in muscle is widely associated with myofibrosis, a condition marked by an elevated presence of fibrous connective tissue along with a diminished ability for muscle regeneration which may cause a gradual replacement of muscle tissue with fibrous connective tissue (Wang and Zhou, 2022). This process is still relatively unknown but elevated levels of myofibrosis appear closely linked with increased perimuscular subcutaneous adipose tissue, leading to a subsequent rise in chronic low-grade inflammation (Zoico et al., 2013). The accumulation of fat and connective tissues in skeletal muscle contribute to significant muscle impairment in older individuals (Correa-de-Araujo and Hadley, 2014) which could potentially contribute to age-related variations in physical performance (Correa-de-Araujo and Hadley, 2014; Correa-de-Araujo et al., 2017). The current research offers a more holistic understanding of age-related transformations within the musculoskeletal system by encompassing not only muscle and fat tissue but also considering muscle quality in relation to connective tissue among older adults.

### Asymmetry indicators and age-related trends

Only a limited number of studies have explored the muscle quality of older adults which included connective tissue using thigh CT scans among older adults (Recenti et al., 2020a; Ricciardi et al., 2020a; Recenti et al., 2021b). Previous study reported that these three tissue types had high correlation with lower extremity functions and BMI (Edmunds et al., 2018; Recenti et al., 2021a). The current study further explored the potential association between asymmetry in the three tissue types, age, and physical function. The results revealed that the asymmetry in muscle and connective tissue width play as major contributors to the variation across different age groups. These results emphasize the potential role of these tissue components in age-related changes and their impact on physical function which is in line with previous research (Zoico et al., 2013; Lee et al., 2019).

Our findings add a new perspective to understanding how different tissue components collectively contribute to functional mobility and overall physical wellbeing by looking at asymmetrical condition in the three types of soft tissues analyzed with NTRA on the mid-tight CT scan. The significant differences observed in all the tissues asymmetry indicators highlight the distinct characteristics of each tissue type. Connective tissue asymmetry’s difference in width reflects its potential involvement in age-related changes underlying the not negligible impact of the connective tissue itself in the sarcopenia studies. Differences in skewness and width in muscle tissue asymmetry suggest alterations in muscle quality, which might be linked to muscle function, mobility, and overall physical performance. The asymmetry in fat tissue amplitude variations across age indicate that adipose tissue plays a pivotal role in age-related changes which reflect changes in body composition, metabolism, and the potential influence of adipose tissue on muscle function with aging (Ribisl et al., 2007). The asymmetry indicators were also able to differentiate levels of BMI among the older Icelandic population of the AGES dataset. The correlation between asymmetry indicators and BMI highlights the potential influence of body composition on tissue distribution and physical function. This could be especially relevant for understanding the impact of obesity on musculoskeletal health and mobility in older adults. This might reflect alterations in body composition, metabolism, and the potential influence of adipose tissue on muscle function (Correa-de-Araujo et al., 2020). Similarly, asymmetry in muscle tissue across age suggests their contribution to changes in musculoskeletal structure and function over time as both weak muscle strength and high BMI is higher associated with poorer physical performance (Hardy et al., 2013). Our findings revealed that the asymmetry indicators in all three tissue types were able to separate normal and slow groups in Fast-GAIT task. The separation of normal and slow groups suggests that asymmetry has the potential to serve as a marker of gait performance and mobility status. They could be utilized to identify individuals who might be at a higher risk of age-related decline in physical function or those who may require tailored interventions to maintain or improve their mobility and overall health. Although TUG tests are well recognized as a predictor of fall or frailty among older individuals (Savva et al., 2013; Barry et al., 2014) the lack of differentiation in asymmetry for the TUG task emphasizes that not all functional tasks may be equally influenced by tissue asymmetry, reflecting the multifaceted nature of physical function. However, incorporating assessments of tissue asymmetry, including muscle, fat, and connective tissue, into clinical evaluations could provide a more nuanced evaluation of muscle health and potential mobility issues in older adults. This approach may allow healthcare professionals to better tailor treatment plans and interventions to address specific tissue-related challenges.

The identification of a relationship between tissue asymmetry and functional mobility suggests new directions for further research. Investigating the underlying mechanisms linking these tissue asymmetries to mobility and exploring the potential influence of different factors including lifestyle, genetics, and exercise could lead to a deeper understanding of musculoskeletal aging processes.

### Limitations and strengths

The strength of the study is the comprehensive analysis on the tissue composition, including muscle, fat, and connective tissue, which provides a holistic understanding of age-related changes. Further, the incorporation of functional mobility tasks including Normal-GAIT, Fast-GAIT, and TUG enhances the relevance of the findings to the real-world physical performance test with muscle quality. The study findings offer potential insights for developing clinical assessment tools that could aid in identifying individuals at risk of functional decline.

The study includes some limitations. First, the cross-sectional nature of the study restricted the ability to establish causal relationships between tissue composition asymmetry and age-related changes or functional performance. Future research could consider incorporating longitudinal designs, which would provide a more comprehensive understanding of the dynamic interplay between tissue asymmetry, aging, and various functional outcomes including comorbidities that typically affect the elderly population. A potential future development could be the employment of machine learning classification algorithms to longitudinally predict comorbidities like cardiac pathophysiology, diabetes, and hypertension utilizing the asymmetry indicators as input including an in-depth feature importance analysis. Second, the study’s sample size might limit the generalizability of findings to broader populations. The current study is a cross-sectional study based secondary analysis and the available data is only for those who completed the physical performance test at the measurement site. Therefore, it is possible that those with available data may have been healthier than those who were excluded or not completed the measurement. Despite the thousands of subjects included in this research, larger and more diverse samples, with a wider range of demographic characteristics and health statuses may further enhance our findings. Thirdly, the study’s asymmetry measurements might not have captured the full complexity of tissue composition and its impact on functional mobility. Utilizing multi-dimensional assessments that encompass factors like muscle strength, flexibility, and coordination could offer a more holistic view for the relationship between tissue asymmetry and functional outcomes in older adults. The study considers limited factors like BMI and gait speed. Future analyses with additional data such as genetics, comorbidities, and lifestyle factors with advanced statistical methods and machine learning predictive approaches might contribute to new insights on the observed outcomes. A further limitation of this study is the use CT scan to analyze old people: CT is typically reserved for specific diagnostic purposes when the benefits outweigh the risks. A recent study demonstrated a significant association between CT scans and blood tumors in young subjects (Bosch de Basea Gomez et al., 2023). This underscores the need for caution in the use of CT scans, prompting a recommendation for further research and clinical exploration to limit their unnecessary application.

Additionally, with a particular reference to the investigated muscle asymmetry indicator (Δσ_mus_), it is worth noting that this reflects the heterogeneity in the muscle tissue, and it may have important implications in the clinical assessment of age-related or pathologic changes in muscle quality (Harris-Love et al., 2019). Therefore, future work will aim at investigating physiological phenomena involved in the tissue asymmetry changes and analyzing other confounding factors through advanced data analysis approaches with aim of defining novel robust quantitative metrics to monitor healthy aging.

## Conclusion

In conclusion, the investigation on asymmetry across various tissue types of muscle, fat, and connective tissue provides a further understanding of the multifaceted factors influencing musculoskeletal health. The significant contributions of the NTRA features in particular muscle and connective tissue width asymmetry indicators to variations across age groups presented their potential roles. These findings suggest that tissue asymmetry could have significant diagnostic and prognostic value as markers for age-related declines and mobility restrictions which provide possibilities for early interventions and personalized treatments. Furthermore, the study’s limitations emphasize the need for future research, including longitudinal studies and more extensive participant samples, to fully elucidate the underlying mechanisms and potential applications of tissue composition asymmetry in aging and physical function. The long-term goal of our research is to define robust quantitative metrics to effectively monitor healthy aging and gain a deeper understanding of the impact of tissue composition on physical function in older adults.

## Data Availability

The datasets presented in this article are not readily available because the AGES I-II dataset cannot be made publicly available, since the informed consent signed by the participants prohibits data sharing on an individual level, as outlined by the study approval by the Icelandic National Bioethics Committee (RU Code of Ethics, Paragraph 3—Article 2—Higher Education Institution Act 63/2006). Requests to access the datasets should be directed to Hjartavernd—The Icelandic Heart Association: afgreidsla@hjarta.is.

## References

[B1] Barry E. Galvin R. Keogh C. Horgan F. Fahey T. (2014). Is the Timed up and Go test a useful predictor of risk of falls in community dwelling older adults: a systematic review and meta-analysis. BMC Geriatr. 14, 14. 10.1186/1471-2318-14-14 24484314 PMC3924230

[B2] Borghi S. Bonato M. La Torre A. Banfi G. Vitale J. A. (2022). Interrelationship among thigh intermuscular adipose tissue, cross-sectional area, muscle strength, and functional mobility in older subjects. Med. Baltim. 101 (26), e29744. 10.1097/MD.0000000000029744 PMC9239645 35777009

[B3] Bosch de Basea Gomez M. Thierry-Chef I. Harbron R. Hauptmann M. Byrnes G. Bernier M. O. (2023). Risk of hematological malignancies from CT radiation exposure in children, adolescents and young adults. Nat. Med. 2023, 1–9. 10.1038/s41591-023-02620-0 PMC10719096 37946058

[B4] Cawthon P. M. (2015). Assessment of lean mass and physical performance in sarcopenia. J. Clin. Densitom. 18 (4), 467–471. 10.1016/j.jocd.2015.05.063 26071168

[B5] Cesari M. Kritchevsky S. B. Penninx B. W. Nicklas B. J. Simonsick E. M. Newman A. B. (2005). Prognostic value of usual gait speed in well-functioning older people—results from the Health, Aging and Body Composition Study. J. Am. Geriatr. Soc. 53, 1675–1680. 10.1111/j.1532-5415.2005.53501.x 16181165

[B6] Chang M. Saczynski J. S. Snaedal J. Bjornsson S. Einarsson B. Garcia M. (2013). Midlife physical activity preserves lower extremity function in older adults: age gene/environment susceptibility-Reykjavik study. J. Am. Geriatr. Soc. 61, 237–242. 10.1111/jgs.12077 23320618 PMC4205047

[B7] Chatterjee P. Pedrini S. Stoops E. Goozee K. Villemagne V. L. Asih P. R. (2021). Plasma glial fibrillary acidic protein is elevated in cognitively normal older adults at risk of Alzheimer’s disease. Transl. Psychiatry 11, 27–10. 10.1038/s41398-020-01137-1 33431793 PMC7801513

[B8] Clark B. C. (2019). Neuromuscular changes with aging and sarcopenia. J. frailty aging 8, 7–9. 10.14283/jfa.2018.35 30734824 PMC9725117

[B9] Correa-de-Araujo R. Addison O. Miljkovic I. Goodpaster B. H. Bergman B. C. Clark R. V. (2020). Myosteatosis in the context of skeletal muscle function deficit: an interdisciplinary workshop at the National Institute on Aging. Front. physiology 11, 963. 10.3389/fphys.2020.00963 PMC7438777 32903666

[B10] Correa-de-Araujo R. Hadley E. (2014). Skeletal muscle function deficit: a new terminology to embrace the evolving concepts of sarcopenia and age-related muscle dysfunction. Journals Gerontology Ser. A Biomed. Sci. Med. Sci. 69 (5), 591–594. 10.1093/gerona/glt208 PMC3999854 24737562

[B11] Correa-de-Araujo R. Harris-Love M. O. Miljkovic I. Fragala M. S. Anthony B. W. Manini T. M. (2017). The need for standardized assessment of muscle quality in skeletal muscle function deficit and other aging-related muscle dysfunctions: a symposium report. Front. physiology 8, 87. 10.3389/fphys.2017.00087 PMC5310167 28261109

[B12] Curtis E. Litwic A. Cooper C. Dennison E. (2015). Determinants of muscle and bone aging. J. Cell. physiology 230 (11), 2618–2625. 10.1002/jcp.25001 PMC4530476 25820482

[B13] Day B. L. Lord S. R. (2018). Balance, gait, and falls. Elsevier.

[B14] Edmunds K. Gíslason M. Sigurðsson S. Guðnason V. Harris T. Carraro U. (2018). Advanced quantitative methods in correlating sarcopenic muscle degeneration with lower extremity function biometrics and comorbidities. PloS one 13 (3), e0193241. 10.1371/journal.pone.0193241 29513690 PMC5841751

[B15] Edmunds K. J. Árnadóttir Í. Gíslason M. K. Carraro U. Gargiulo P. (2016). Nonlinear trimodal regression analysis of radiodensitometric distributions to quantify sarcopenic and sequelae muscle degeneration. Comput. Math. Methods Med. 2016, 1–10. 10.1155/2016/8932950 PMC5223076 28115982

[B16] Edmunds K. J. Okonkwo O. C. Sigurdsson S. Lose S. R. Gudnason V. Carraro U. (2021). Soft tissue radiodensity parameters mediate the relationship between self-reported physical activity and lower extremity function in AGES-Reykjavík participants. Sci. Rep. 11 (1), 20173. 10.1038/s41598-021-99699-7 34635746 PMC8505499

[B17] Gonzalez M. Gates D. H. Rosenblatt N. J. (2020). The impact of obesity on gait stability in older adults. J. Biomech. 100, 109585. 10.1016/j.jbiomech.2019.109585 31911052 PMC7061260

[B18] Goodpaster B. H. Park S. W. Harris T. B. Kritchevsky S. B. Nevitt M. Schwartz A. V. (2006). The loss of skeletal muscle strength, mass, and quality in older adults: the health, aging and body composition study. J. Gerontol. A Biol. Sci. Med. Sci. 61, 1059–1064. 10.1093/gerona/61.10.1059 17077199

[B19] Guralnik J. M. Ferrucci L. Pieper C. F. Leveille S. G. Markides K. S. Ostir G. V. (2000). Lower extremity function and subsequent disability consistency across studies, predictive models, and value of gait speed alone compared with the short physical performance battery. J. Gerontol. A Biol. Sci. Med. Sci. 55, M221–M231. 10.1093/gerona/55.4.M221 10811152 PMC12149745

[B20] Hardy R. Cooper R. Aihie Sayer A. Ben-Shlomo Y. Cooper C. Deary I. J. (2013). Body mass index, muscle strength and physical performance in older adults from eight cohort studies: the HALCyon programme. PloS one 8 (2), e56483. 10.1371/journal.pone.0056483 23437142 PMC3577921

[B21] Harris T. B. Launer L. J. Eiriksdottir G. Kjartansson O. Jonsson P. V. Sigurdsson G. (2007). Age, gene/environment susceptibility–Reykjavik study: multidisciplinary applied phenomics. Amer. J. Epidemiol. 165 (9), 1076–1087. 10.1093/aje/kwk115 17351290 PMC2723948

[B22] Harris‐Love M. O. Gonzales T. I. Wei Q. Ismail C. Zabal J. Woletz P. (2019). Association between muscle strength and modeling estimates of muscle tissue heterogeneity in young and old adults. J. Ultrasound Med. 38 (7), 1757–1768. 10.1002/jum.14864 30548644 PMC9003580

[B23] Kalseth J. Halvorsen T. (2020). Health and care service utilisation and cost over the life-span: a descriptive analysis of population data. BMC Health Serv. Res. 20, 435. 10.1186/s12913-020-05295-2 32429985 PMC7236310

[B24] Kalyani R. R. Corriere M. Ferrucci L. (2014). Age-related and disease-related muscle loss: the effect of diabetes, obesity, and other diseases. lancet Diabetes and Endocrinol. 2 (10), 819–829. 10.1016/s2213-8587(14)70034-8 24731660 PMC4156923

[B25] Khaleghi M. M. Emamat H. Marzban M. Farhadi A. Jamshidi A. Ghasemi N. (2023). The association of body composition and fat distribution with dysmobility syndrome in community-dwelling older adults: bushehr Elderly Health (BEH) program. BMC Musculoskelet. Disord. 24 (1), 809. 10.1186/s12891-023-06934-5 37828473 PMC10568758

[B26] Laroche D. P. Cook S. B. Mackala K. (2012). Strength asymmetry increases gait asymmetry and variability in older women. Med. Sci. Sports Exerc 44, 2172–2181. 10.1249/MSS.0b013e31825e1d31 22617401 PMC3463648

[B27] Lee E. J. Lee S. A. Soh Y. Kim Y. Won C. W. Chon J. (2019). Association between asymmetry in lower extremity lean mass and functional mobility in older adults living in the community. Med. Baltim. 98, e17882. 10.1097/MD.0000000000017882 PMC6855585 31702661

[B28] Mertz K. H. Reitelseder S. Jensen M. Lindberg J. Hjulmand M. Schucany A. (2019). Influence of between-limb asymmetry in muscle mass, strength, and power on functional capacity in healthy older adults. Scand. J. Med. Sci. Sports 29, 1901–1908. 10.1111/sms.13524 31353627

[B29] Miller R. R. Eastlack M. Hicks G. E. Alley D. E. Shardell M. D. Orwig D. L. (2015). Asymmetry in CT scan measures of thigh muscle 2 Months after hip fracture: the Baltimore hip studies. J. Gerontol. Ser. A 70, 1276–1280. 10.1093/gerona/glv053 PMC4612358 25969469

[B30] Nakakubo S. Doi T. Makizako H. Tsutsumimoto K. Hotta R. Kurita S. (2018). Association of walk ratio during normal gait speed and fall in community-dwelling elderly people. Gait posture 66, 151–154. 10.1016/j.gaitpost.2018.08.030 30195217

[B31] Newton R. A. (1997). Balance screening of an inner city older adult population. Arch. Phys. Med. Rehabil. 78, 587–591. 10.1016/s0003-9993(97)90423-8 9196465

[B32] Ostir G. V. Berges I. M. Ottenbacher K. J. Fisher S. R. Barr E. Hebel J. R. (2015). Gait speed and dismobility in older adults. Arch. Phys. Med. Rehabil. 96, 1641–1645. 10.1016/j.apmr.2015.05.017 26067366

[B33] Perry M. C. Carville S. F. Smith I. C. H. Rutherford O. M. Newham D. J. (2007). Strength, power output and symmetry of leg muscles: effect of age and history of falling. Eur. J. Appl. physiology 100, 553–561. 10.1007/s00421-006-0247-0 16847676

[B34] Podsiadlo D. Richardson S. (1991). The timed “Up and Go”: a test of basic functional mobility for frail elderly persons. J. Am. Geriatr. Soc. 39, 142–148. 10.1111/j.1532-5415.1991.tb01616.x 1991946

[B35] Recenti M. Ricciardi C. Edmunds K. Gislason M. K. Gargiulo P. (2020b). Machine learning predictive system based upon radiodensitometric distributions from mid-thigh CT images. Eur. J. Transl. Myology 30 (1), 121–124. 10.4081/ejtm.2019.8892 PMC7254455 32499893

[B36] Recenti M. Ricciardi C. Edmunds K. Jacob D. Gambacorta M. Gargiulo P. (2021b). Testing soft tissue radiodensity parameters interplay with age and self-reported physical activity. Eur. J. Transl. myology 31 (3), 9929. 10.4081/ejtm.2021.9929 PMC8495362 34251162

[B37] Recenti M. Ricciardi C. Edmunds K. J. Gislason M. K. Sigurdsson S. Carraro U. (2020a). Healthy aging within an image: using muscle radiodensitometry and lifestyle factors to predict diabetes and hypertension. IEEE J. Biomed. Health Inf. 25 (6), 2103–2112. 10.1109/jbhi.2020.3044158 33306475

[B38] Recenti M. Ricciardi C. Monet A. Jacob D. Ramos J. Gìslason M. (2021a). Predicting body mass index and isometric leg strength using soft tissue distributions from computed tomography scans. Health Technol. 11, 239–249. 10.1007/s12553-020-00498-3

[B39] Reinders I. Murphy R. A. Koster A. Brouwer I. A. Visser M. Garcia M. E. (2015). Muscle quality and muscle fat infiltration in relation to incident mobility disability and gait speed decline: the age, gene/environment susceptibility-reykjavik study. J. Gerontol. A Biol. Sci. Med. Sci. 70, 1030–1036. 10.1093/gerona/glv016 25748031 PMC4506318

[B40] Ribisl P. M. Lang W. Jaramillo S. A. Jakicic J. M. Stewart K. J. Bahnson J. (2007). Exercise capacity and cardiovascular/metabolic characteristics of overweight and obese individuals with type 2 diabetes: the Look AHEAD clinical trial. Diabetes care 30 (10), 2679–2684. 10.2337/dc06-2487 17644623

[B41] Ricciardi C. Edmunds K. J. Recenti M. Sigurdsson S. Gudnason V. Carraro U. (2020a). Assessing cardiovascular risks from a mid-thigh CT image: a tree-based machine learning approach using radiodensitometric distributions. Sci. Rep. 10 (1), 2863. 10.1038/s41598-020-59873-9 32071412 PMC7029006

[B42] Savva G. M. Donoghue O. A. Horgan F. O’Regan C. Cronin H. Kenny R. A. (2013). Using timed up-and-go to identify frail members of the older population. J. Gerontol. Ser. A 68 (4), 441–446. 10.1093/gerona/gls190 22987796

[B43] Schaap L. A. Koster A. Visser M. (2013). Adiposity, muscle mass, and muscle strength in relation to functional decline in older persons. Epidemiol. Rev. 35, 51–65. 10.1093/epirev/mxs006 23221972

[B44] Simonsick E. M. Newman A. B. Visser M. Goodpaster B. Kritchevsky S. B. Rubin S. (2008). Mobility limitation in self-described well-functioning older adults: importance of endurance walk testing. J. Gerontol. A Biol. Sci. Med. Sci. 63 (8), 841–847. 10.1093/gerona/63.8.841 18772472 PMC5722013

[B45] Skelton D. A. Kennedy J. Rutherford O. M. (2002). Explosive power and asymmetry in leg muscle function in frequent fallers and non‐fallers aged over 65. Age ageing 31 (2), 119–125. 10.1093/ageing/31.2.119 11937474

[B46] Stagi S. Moroni A. Micheletti Cremasco M. Marini E. (2021). Body composition symmetry in long-term active middle-aged and older individuals. Int. J. Environ. Res. Public Health 18, 5956. 10.3390/ijerph18115956 34199340 PMC8199499

[B47] Studenski S. Perera S. Patel K. (2011). Gait speed and survival in older adults. JAMA 305 (1), 50–58. 10.1001/jama.2010.1923 21205966 PMC3080184

[B48] Treacy D. Hassett L. Schurr K. Fairhall N. J. Cameron I. D. Sherrington C. (2022). Mobility training for increasing mobility and functioning in older people with frailty. Cochrane Database Syst. Rev. 2022, CD010494. 10.1002/14651858.CD010494.pub2 PMC9245897 35771806

[B49] Visser M. Goodpaster B. H. Kritchevsky S. B. Newman A. B. Nevitt M. Rubin S. M. (2005). Muscle mass, muscle strength, and muscle fat infiltration as predictors of incident mobility limitations in well-functioning older persons. J. Gerontol. A Biol. Sci. Med. Sci. 60, 324–333. 10.1093/gerona/60.3.324 15860469

[B50] Wang X. Zhou L. (2022). The many roles of macrophages in skeletal muscle injury and repair. Front. Cell Dev. Biol. 10, 952249. 10.3389/fcell.2022.952249 35898401 PMC9309511

[B51] White D. K. Neogi T. Nevitt M. C. Peloquin C. E. Zhu Y. Boudreau R. M. (2013). Trajectories of gait speed predict mortality in well-functioning older adults: the Health, Aging and Body Composition study. J. Gerontol. A Biol. Sci. Med. Sci. 68 (4), 456–464. 10.1093/gerona/gls197 23051974 PMC3593620

[B52] WHO (2020). UN decade of healthy ageing 2021-2030. Available at: https://www.who.int/initiatives/decade-of-healthy-ageing (Accessed August 8, 2023).

[B53] WHO (2021). Obesity and overweight (WHO fact sheet No. 311). Available at: https://www.who.int/news-room/fact-sheets/detail/obesity-and-overweight (Accessed August 23, 2023).

[B54] Wilkinson D. J. Piasecki M. Atherton P. J. (2018). The age-related loss of skeletal muscle mass and function: measurement and physiology of muscle fibre atrophy and muscle fibre loss in humans. Ageing Res. Rev. 47, 123–132. 10.1016/j.arr.2018.07.005 30048806 PMC6202460

[B55] Young A. Stokes M. Crowe M. (1985). The size and strength of the quadriceps muscles of old. Clin. Physiol. Oxf. Engl. 5 (2), 145–154. 10.1111/j.1475-097x.1985.tb00590.x 3888498

[B56] Zoico E. Corzato F. Bambace C. Rossi A. P. Micciolo R. Cinti S. (2013). Myosteatosis and myofibrosis: relationship with aging, inflammation and insulin resistance. Archives gerontology geriatrics 57 (3), 411–416. 10.1016/j.archger.2013.06.001 PMC5278642 23809667

